# The Races of Men—A Fragment

**Published:** 1850-12

**Authors:** 


*The Races of Men.—A Fragment.* By Robert Knox, M. D., Lecturer on Anatomy, and Corresponding Member of the National Academy of Medicine of France. 8vo. pp. 323. American edition. Philadelphia, 1850.

The author of this unique production may be known to many of our readers, if for no other effort, for his translation of the Human Anatomy of H. Cloquet. He has, however, made some little noise in the world as a lecturer on the subjects embraced in the volume before us; and by the promulgation of opinions on those subjects, which are certainly sufficiently wild and startling, and on which he appears to pride himself not a little. In speaking of his dogma, that "human character, individual and national, is traceable solely to the nature of that race to which the individual or nation belongs," he characterizes it, very properly we think, as a statement which must meet with the severest opposition. "It runs counter to nearly all the chronicles of events, called histories,"—a sorry recommendation to favor we should say,—"it shocks the theories of statesmen, theologians, philanthropists of all shades; nevertheless, it is simply a fact, the most remarkable, the most comprehensive, which philosophy has announced. Race is everything: literature, science, art, in a word, civilization, depend on it."

Such is a specimen of the author's manner, and it is no exaggeration to say, that the whole work abounds with similar categorical affirmations,—at times plausible; at others revolting; occasionally supported on an admitted basis; at others altogether aerial; and not unfrequently in contrast with all fact, observation, and even common sense; strung together, too, in the most confused and discordant manner, and presenting

> "A fine specimen, on the whole,
> Of what the learned call rigmarole."

It is difficult, indeed, to classify such a production. The author himself denominates it "a fragment." Its fragmentary character cannot be questioned, if the word be used to signify "an imperfect part;" but were we to assign it a place, we should say it belongs rather to another class of productions, and is more deserving of the title "The Races of Men—a *rhapsody;*" not in the ancient and favorable sense of the word as applicable to Homeric periods; but in its more modern acceptation, "a confused jumble of sentences or statements, without dependance or natural connection, a rambling composition." But even in this point of view the work might have possessed a claim to respectability, were it not grossly disfigured with general and special references to persons and things, couched in language often—it is true—forcible, but not unfrequently of the most offensive character. What apology, for example, can be offered for the following scurrilous allusion to one of the most gifted writers of this country.

"Buffon concluded that animal life was not so vigorous on the American soil as in the old world, comparing one animal with another; this simple fact, for it is one, roused the wrath of an Anglo-Saxon, now settled in that country, but calling himself an American; I mean Mr. Cooper, the novelist. True to his Saxon race, he has determined to make out, in the face of all common sense and truth—despising the one by his trade or calling, and being seemingly without the other—that the American soil nourished as big animals as ever were grown in old France or England, or the whole world; that the buffalo was as large as our oxen, and the turkey larger than the barn-door fowl; what a pity he had not also added, that geese and asses of all kinds abound, and are at least, as large as pedantic, and as stupidly solemn as any the Britishers could ever boast of. This is the Mr. Cooper who compared, through ten drawlishly-spun pages, the Rhine with the immortal Hudson—the everlasting Hudson—that large river which runs near the ancient city of New York, so rich in the association of great names and stirring events. What solemn pedantry, what deplorable want taste and sense, to forget the passage of the Rhine by Cæsar and Napoleon! These are the names which give immortality to the Rhine, not the amount of water it contains, nor its length nor breadth; it is not the size of the Nile which makes it live in the recollection of nations. Do you not see in this miserable comparison of Mr. Cooper the egotism of the Saxon peep out in all its true colors? Our rivers are bigger than yours—prettier, deeper; our horses are faster than yours—fatter and better; our oxen are larger than yours—sleeker and finer. You will excuse, I trust, these critical remarks; folly and egotism require severe censure, whether individual or national—in fact, these terms are identical, nations merely being aggregates of individuals."—p. 167.

The *modesty* of the author is characteristically exhibited throughout the work. "Materials," he says, "for a systematic history of the races of men are wholly wanting; the great problem of human nature has scarcely been touched on in any previous history of race." And again:—"Physiologists will dispute with me the great law I have endeavored to substitute for the effete common place of the schools; the geologists will think me hasty in declaring the era of Cuvier at an end; the theologian—but here I stop; a reply shall not be wanting. As to hack compilers, their course is simple: they will first deny the doctrine to be true; when this becomes clearly untenable, they will deny that it is new, and they will finish by engrossing the whole in their next compilations, omitting carefully the name of the author."

There are two or three points in this last quotation that demand comment. The most ardent stickler for originality, will not deny the claims of the author to it, both on the score of bold assertion, and novelty of expression; but whilst we admit this, there are few, we think, who can or will accord with him. The world are apt to ascribe great merit to the propounder of original views, no matter how extravagant and ephemeral they may be; whilst the sober, steady pursuer of science, who day by day unostentatiously developes the laws of phenomena, may receive but a feeble tribute of praise; yet the one may be—as in the present case—a visionary enthusiast—an *original*—whilst the other has all the endowments of the real philosopher. The Author need not, we feel satisfied, be alarmed at his views being wholly "engrossed" in any compilation; and if his name be carefully omitted by writers on the subject, it will only be because his speculations have themselves not been deemed worthy of notice.

But let us see how he deals with others, and how, he himself disposes of facts that do not square with his ideas.

"I have always, he remarks, doubted the fact of cannibalism having ever existed. A patient inquiry into the history of the American race satisfied me that the cannibalism of the New world was the pure invention of the Catholic missionaries: the cannibalism of the East may, I think, be traced to a similar source. I never met with any one who had been present at such a banquet. In Africa no such practice exists. The whole affair, I think, a romance, but it has served its purpose with those who think that the end vindicates the means."—p. 316.

Yet it is no romance. A scientific friend attached to the United States exploring expedition, assured us, that he has seen the Feejeeman gnawing a human bone; and the authentic records of that expedition are fatal to all of Dr. Knox's conclusions on the subject.

The fragmentary character of his speculations on the races of men may in part be appreciated by the following extract.

"The races of men as they now exist on the globe constitute a fact which cannot be overlooked. They differ from each other widely—most widely :—but that such differences exist, and important ones too, has not been denied; the word, *race*, is of daily use, applied even to man; since the war of race commenced in continental Europe and in Ireland, no expression is of more frequent occurrence than the term race. It is not, then a new phrase I use, but I use it in a new sense; for whilst the statesman, the historian, the theologian, the universalist, and the mere scholar, either attached no special meaning to the term, for reasons best known to themselves; or refused to follow out the principle to its consequences; or ascribed the moral difference in the races of men to fanciful causes, such as education, religion, climate, &c.,—and their physical distinctions sometimes to the same hap-hazard influences—sometimes to climate alone—sometimes to climate aided by a mysterious law—such as that imagined by Prichard, that the fair individuals of any family separating themselves from the darker branches would with each successive generation become fairer, and the darker become darker, forgetting that this theory was refuted by the very first fact from which he starts, and which actually forms the basis of his whole theory—namely, that individuals having a specific tendency towards different races are constantly being born in every family ;—or, lastly, ascribing to mere chance and hap-hazard, as in the story of the short-legged American sheep, the production of the permanent varieties of man :—I, in opposition to these views, am prepared to assert that race is everything in human history; that the races of men are not the result of accident; that they are not convertible into each other by any contrivance whatever. The eternal laws of nature must prevail over protocols and dynasties: fraud,—that is, the law; and brute force—that is, the bayonet, may effect much; have effected much; but they cannot alter nature."—p. 13.

And again:

"What is race, and what is species? These terms are easier understood than defined. That the idea of distinct species and of race is fast passing away from the human mind, may or may not be true; the old doctrine has been deeply shaken; still species and race exist for us; for man at least; in space, though not in time. In time there is probably no such thing as species: no absolutely new creations ever took place; but, as viewed by the limited mind of man, the question takes another aspect. As regards his individual existence, time is a short span; a few centuries or a few thousand years, more or less: this is all he can grasp. Now, for that period at least, organic forms seem not to have changed. So far back as history goes, the species of animals as we call them have not changed; the races of men have been absolutely the same. They were distinct then for that period as at present. Are they commutable into each other? Are these causes in constant operation, slowly yet surely altering and changing everything? Or does this happen by sudden cataclasms or geological epochs? Of one thing we are certain, entire races of animals have disappeared from the surface of the globe; other seemingly new creations occupy their place. But is it really a new creation? This question we shall also discuss.

Look more narrowly into the races of men, and you will find them to be subject to diseases peculiar to each; that the very essence of their language is distinct; their civilization also, if they have any. Trace the matter further, and you will find that transcendental anatomy can alone explain these mysterious circumstances: how all embryos should resemble each other; how they should resemble the primitive forms of life when the world was yet young; how deviations in form or varieties, not intended to be permanent, should repeat primitive forms, as proved by fossil remains; or present human or bestial forms; or take unknown shapes, referring, no doubt, to the future: lastly, and that is the most difficult question, how specializations should ever appear at all, and be, for a time at least, permanent. Two questions remain, beyond, I fear, human enquiry:—1st. The origin of life on the globe; 2d. The secondary laws, for they must be so, and can be nothing else, which create out of primitive forms, the past, the present, and the future organic worlds, clothing them with beauteous scenery. Endless, but defined variety of forms, adorn the earth, the air, the waters; the scheme of creation, in fact, so far as man's feeble reason can judge; not the object of creation; not the object of man's creation, which, though wonderful, is not more so than that of any other form; not then the object of man's creation as an intellectual being; this has been revealed to us by divine minds. But I must view this last question also as an anatomist and physiologist, confining my remarks to man merely as a material being; the most perfect, no doubt, that exists. In woman's form I see the perfection of Nature's works; the absolutely perfect; the beautiful, the highest manifestation of abstract life, clothed in a physical form, adapted to the corresponding minds of her race and species."—p. 34.

In regard to the origin of man, we have the following vague apostrophe:—

"The origin of man is a myth, which each race interprets in its own way, formules after the fashion of its own intellectual bearing; retouches as it makes progress, in arts, literature, and science; that is, in civilization.

I mean not here to discuss these myths. The Jewish myth seems to have been a purely material one; philosophic, and sublimely simple, it offers no details. The Coptic and Hindoo was spiritual and lofty, but debased by shocking obscenities; the minds of the races were not equal to the perception of the perfect and the beautiful. The Scandinavian myth was coarse and brutal; material in its essence: the hideous representations of the Deity in India, China, Mongolia, and Polynesia, indicate the sad character of the minds of these races.

The precise geological period when man appeared on the earth, has not been determined; nor what race appeared first; nor under what form. But it is evident that man has survived several geological eras. On these points all is at present conjecture; but as man merely forms a portion of the material world, he must of necessity be subject to all the physiological and physical laws affecting life on the globe. His pretensions to place himself above nature's laws, assume a variety of shapes: sometimes he affects mystery; at other times he is grandly mechanical. Now, all is to be done through the workshop, in a little while, the ultimatum (what is the ultimatum aimed at?) is to be gained through religion: and thus man frets his hour upon the stage of life, fancying himself something whilst he is absolutely nothing. For him worlds were made millions of years ago, and yet according to his own account he appeared, as it were, but yesterday. Let us leave human chronology to the chronicler of events; it turned the brain of Newton."—p. 323.

His speculations in regard to the various races of man, are singularly fantastic; but we have no space, even had we the inclination to follow him. It may be sufficient to cite his—what he admits to be—"brief and hasty and imperfect sketch of the dark races."

"No one seems much to care for them. Their ultimate expulsion from all lands which the fair races can colonize seems almost certain. Within the tropic, climate comes to the rescue of those whom Nature made, and whom the white man strives to destroy; each race of white men after their own fashion: the Celt, by the sword; the Saxon, by conventions, treaties, parchment, law. The result is ever the same—the robbing the colored races of their lands and liberty. Thirty years ago a military *rhazia*, composed of English soldiers, Dutch boors, and native Hottentots, devastatated the beautiful territory of the Amakoso Kaffirs. We reached the banks of the Kei, and the country of the noble Hinsa, where wandered the 'wilde' of Nature's creation. All must disappear shortly before the rude civilization of the Saxon boor—antelope and hippopotamus, giraffe and Kaffir."—p. 210.

And he elsewhere adds:

"If there be a dark race destined to contend with the fair races of men for a portion of the earth, given to man as an inheritance, it is the Negro. The tropical regions of the earth seem peculiarly to belong to him; his energy is considerable: aided by a tropical sun, he repels the white invader. From St. Domingo he drove out the Celt; from Jamaica he will expel the Saxon; and the expulsion of the Lusitanian from Brazil, by the Negro, is merely an affair of time."—p. 306.

Should the "fragment" ever be followed by another chip of the same block, we trust that the author will endeavor to be guided by more system in his mode of expounding his views. He appears, indecd, to have been impressed with the imperfect order in which the "lectures" before us have been arranged. "The greatest difficulty," he says, "I have experienced in the drawing up of these lectures, whether as lectures delivered to public audiences, or written as they now are for publication, has been to decide on the arrangement best calculated to submit my views briefly, yet intelligibly, to the public. After various trials I have decided on the following: it may not be the best; it is not systematic; it is not methodical; but it seems to me adapted to a very numerous class of readers, who, though highly educated, are yet not scientific. To place the great physiological principles regulating human and other living beings before them in an intelligible form, has been of course my main difficulty;" (p. 25) And again, "In presenting the first complete edition of my Lectures on the Races of Men to public criticism, I have weighed most anxiously the form of publication, and the order or method to be followed in arranging the lectures. It has, indeed, been my great difficulty."

